# Uncovering plant-pathogen crosstalk through apoplastic proteomic studies

**DOI:** 10.3389/fpls.2014.00249

**Published:** 2014-06-03

**Authors:** Bertrand Delaunois, Philippe Jeandet, Christophe Clément, Fabienne Baillieul, Stéphan Dorey, Sylvain Cordelier

**Affiliations:** Laboratoire Stress, Défenses et Reproduction des Plantes, Unité de Recherche Vignes et Vins de Champagne-EA 4707, Université de Reims Champagne-ArdenneReims, France

**Keywords:** apoplast, cell wall, proteomics, secretome, pathogen, defense, MAMP

## Abstract

Plant pathogens have evolved by developing different strategies to infect their host, which in turn have elaborated immune responses to counter the pathogen invasion. The apoplast, including the cell wall and extracellular space outside the plasma membrane, is one of the first compartments where pathogen-host interaction occurs. The plant cell wall is composed of a complex network of polysaccharides polymers and glycoproteins and serves as a natural physical barrier against pathogen invasion. The apoplastic fluid, circulating through the cell wall and intercellular spaces, provides a means for delivering molecules and facilitating intercellular communications. Some plant-pathogen interactions lead to plant cell wall degradation allowing pathogens to penetrate into the cells. In turn, the plant immune system recognizes microbial- or damage-associated molecular patterns (MAMPs or DAMPs) and initiates a set of basal immune responses, including the strengthening of the plant cell wall. The establishment of defense requires the regulation of a wide variety of proteins that are involved at different levels, from receptor perception of the pathogen via signaling mechanisms to the strengthening of the cell wall or degradation of the pathogen itself. A fine regulation of apoplastic proteins is therefore essential for rapid and effective pathogen perception and for maintaining cell wall integrity. This review aims to provide insight into analyses using proteomic approaches of the apoplast to highlight the modulation of the apoplastic protein patterns during pathogen infection and to unravel the key players involved in plant-pathogen interaction.

## Introduction

Higher plants interact continuously with microbes such as viruses, bacteria, oomycetes or fungi, some of which are phytopathogens, leading to plant diseases. The lifestyle of the pathogen determines the nature of this interaction (Doehlemann and Hemetsberger, 2013). Biotrophic pathogens have developed specific strategies to interact with the cell wall and keep plant cells alive during their life cycles. In contrast, necrotrophic pathogens feed on dead plant cells. Plants naturally display preformed defenses, which include the cell wall and cuticle acting as a physical barrier. However, these preformed defenses are sometimes not strong enough to stop the invading pathogen. Successful resistance then comes from a rapid activation of the plant's innate immune system (Boller and Felix, 2009). Plant perception of conserved molecules characteristic of many microbes is the first step in this innate immune response. These molecules, more commonly called general elicitors, are also referred to microbe-associated molecular patterns (MAMPs) (Jones and Dangl, 2006; Pel and Pieterse, 2013). MAMPs are recognized by pattern recognition receptors (PRRs), which are generally localized at the level of the plasma membrane. MAMP perception leads to the establishment of the so-called MAMP-triggered immunity (MTI) (Boller and Felix, 2009). The small peptide flg22 derived from bacterial flagellin and the elongation-factor Tu peptide elf18 are the most extensively studied MAMPs (Felix and Boller, 2003; Zipfel, 2009; Trdá et al., 2013). Many other MAMPs have been identified (reviewed in Newman et al., 2013) such as eicosapolyenoic acids (Bostock et al., 1981; Savchenko et al., 2010), β-glucans (Umemoto et al., 1997; Klarzynski et al., 2000), peptidoglycans (Willmann et al., 2011), lipopolysaccharides (Newman et al., 2002; Erbs and Newman, 2012), rhamnolipids (Varnier et al., 2009; Sanchez et al., 2012), or chitin oligomers (Kaku et al., 2006; Miya et al., 2007). Pathogens can suppress MTI by secreting effector proteins that act either by inhibiting the MAMP-PRR interaction or downstream signaling. The direct or indirect recognition of effectors (previously called specific elicitors) by plant resistance gene products results in the so-called effector-triggered immunity (ETI) (Pel and Pieterse, 2013). ETI is usually quantitatively stronger than MTI and associated with more sustained and robust immune responses including localized cell death (HR, hypersensitive response) (Tsuda and Katagiri, 2010). Some plant-derived molecules called damage-associated molecular patterns (DAMPs) are also recognized by the plant itself and can trigger an immune response (Boller and Felix, 2009). The well-known systemin or oligogalacturonides released upon cell wall damage were shown to act as DAMPs (Pearce et al., 1991; Schweizer et al., 1996; Denoux et al., 2008; Brutus et al., 2010; Ferrari et al., 2013). Recently, the plant endogenous peptides AtPeps have also been characterized as powerful DAMPs (Yamaguchi and Huffaker, 2011).

Induction of defenses by MAMPs, DAMPS or effectors starts within minutes after signal perception with ion fluxes, MAPK kinase activation, the production of reactive oxygen species (ROS) and reactive nitrogen species (RNS) such as nitric oxide (NO) (Garcia-Brugger et al., 2006; Scheler et al., 2013). ROS and NO can act in signaling and have direct antimicrobial effects. ROS are also involved in plant cell wall strengthening by oxidative cross-linking of polymers. During the plant immune response, the phytohormones salicylic acid (SA), jasmonic acid (JA), and ethylene (ET) play a key role in signal transduction (Robert-Seilaniantz et al., 2011). The importance of JA and SA as primary signals in the regulation of plant immune responses has been well established (Robert-Seilaniantz et al., 2011; Pieterse et al., 2012). The JA pathway is primarily induced by and effective in mediating resistance against necrotrophic pathogens, whereas the SA pathway is primarily induced by and effective in mediating resistance against biotrophic pathogens (Glazebrook, 2005). Nonetheless, this is an over-simplistic view of disease resistance mechanisms as there are complex repertoires of plant hormones that play a role in defense signaling pathways. Indeed, other hormones such as auxin, abscisic acid, cytokinins, and brassinosteroids function as modulators of the plant immune signaling network (Robert-Seilaniantz et al., 2011; Pieterse et al., 2012). Not surprisingly, pathogenic microbes have developed strategies to manipulate plant hormonal pathways in order to divert the immune signaling mechanism for their own benefit. Recent studies suggest that these pathogen-induced modulations of signaling pathways via hormones contribute to virulence (Pieterse et al., 2012). Therefore, the complex crosstalk and induced hormonal changes modulate disease and resistance with the outcomes dependent on pathogen lifestyles and the genetic constitution of the host (Robert-Seilaniantz et al., 2011; Gimenez-Ibanez and Solano, 2013). Thus, pathogens have adapted different types of complex interactions. Biotrophs depend on the living plant metabolism as their nutritional source, and therefore interact intimately with the host cells to modify metabolic processes (Glazebrook, 2005; Horbach et al., 2011). Necrotrophs, on the other hand, invade and kill the plant tissue, feeding on the dead tissue debris. To this end, they usually macerate plant tissues by secreting toxins and abundant hydrolytic enzymes that degrade cell wall polymers (Laluk and Mengiste, 2010). As for the plant, defense responses occur upon pathogen recognition through reinforcement of the plant cell wall to counter pathogen invasion, while production of antimicrobial compounds such as phytoalexins (Jeandet et al., 2013) and synthesis of pathogenesis-related (PR) proteins including hydrolytic enzymes like β-1,3-glucanases and chitinases (Van Loon et al., 2006) contribute to the alteration of pathogen integrity.

Studies of plant-pathogen interactions are numerous in the literature and include a wide range of physiological, molecular and biochemical approaches. Proteomics has become an important tool for large-scale analysis of the proteins involved in the complex plant-pathogen interactions in the post-genomic area (for review see Quirino et al., 2010; Jayaraman et al., 2012). Characterization of a set of proteins under specific plant-pathogen interactions provides a more direct view of cellular processes than DNA or RNA analysis. Proteomics provides insight into protein localization, protein–protein interactions, enzymatic complexes or post-translational modifications that are essential to a better understanding of plant-pathogen interactions. Proteomic approaches have been used in recent years to further characterize plant interactions with viruses (Casado-Vela et al., 2006; Giribaldi et al., 2011; Li et al., 2011; Di Carli et al., 2012), bacteria (Jones et al., 2006; Afroz et al., 2009, 2013; Li et al., 2012), or fungi (Kim et al., 2004a; Geddes et al., 2008; Bhadauria et al., 2010; Mukherjee et al., 2010; Shah et al., 2012). The general picture of changes occurring in the plant-host proteome highlights common features among the broad range of interaction analyzed. A common response, observed in almost all studies, is related to plant photosynthetic activity, which is negatively regulated by pathogen infection, most probably reflecting allocation of energy resources to a general plant defense regulatory mechanism. In parallel, plants counteract to pathogen infection by modulating the accumulation of defense- or stress-associated proteins and proteins involved in ROS metabolism. However, most of the key proteins involved in the plant-pathogen interaction are probably produced at low levels and the majority of studies only detected the most abundant pathogen protein, such as coat protein for virus. These global approaches also present significant technical challenges, as they generally need to differentiate between plant and pathogen proteins (Mathesius, 2009). Simplified models have been developed to circumvent these technical challenges. In some studies, the plant was treated with a MAMP from a pathogen (Chivasa et al., 2006; Liao et al., 2009) or with a signal molecule (Rajjou et al., 2006; Macarisin et al., 2009) to characterize the proteomic changes within the plant. Other studies focused on the pathogen secretome alone (Brown et al., 2012; Girard et al., 2013) or in the presence of plant extracts (Phalip et al., 2005; Fernandez Acero et al., 2009). However, the absence of one of the actors in these simplified models underestimates the complexity of the events occurring during the plant-pathogen crosstalk. The large dynamic range of protein abundance present in plant-pathogen samples, such as pathogen-infected leaves represents an additional difficulty (Bindschedler and Cramer, 2011). In fact, many important proteins are present at low level and are thus difficult to isolate from complex mixtures containing more highly abundant proteins. As the resolution of analytical separation methods is too limited to dissect the total proteome of a cell or a tissue, less abundant proteins are often masked by those produced at higher levels. Sub-cellular proteomics has the advantage not only of relating proteins to a functional compartment of eukaryotic cells, but also of reducing the complexity of the whole cell or tissue protein extracts (Brunet et al., 2003). However, the isolation of sub-cellular proteins typically requires a series of labor-intensive steps. Thus, efficient protocols for sub-cellular fractionation, purification, and enrichment are necessary for each cellular compartment (Lee et al., 2013).

Important processes such as development, intercellular communications or defense mechanisms take place in the apoplast (Sakurai, 1998). The apoplastic proteins are involved in different physiological and biological processes related to growth regulation, biotic and abiotic stresses and cell wall maintenance (Ellis et al., 2007; Tseng et al., 2009). The apoplast or apoplastic space is one of the first physiological compartments of pathogen-host exchanges and the key processes that occur there during microbial infections therefore determine the fate of the interaction (Doehlemann and Hemetsberger, 2013). The apoplast is defined as the extracellular matrix or plant cell wall and the intercellular spaces where the apoplastic fluid circulates (Agrawal et al., 2010). The apoplastic fluid plays a key role in intercellular and intracellular communications and is composed of many substances, notably nutrients, polysaccharides, secondary metabolites and secreted proteins. Lohaus et al. (2001) showed low metabolite concentrations in the apoplastic solution from *Vicia faba*, *Spinacia oleracea*, *Hordeum vulgare*, and *Zea mays*. The sucrose concentration was about 1–2 mM in all plant species, whereas the concentration of hexoses differed strongly between the species. Similarly, the highest concentration of amino acids in the apoplastic solution was found in *Vicia faba* (about 10 mM), the lowest in *Zea mays* (about 2 mM). It is also well known that the redox and pH control in the apoplast serves as a mechanism to respond to environmental signals. The main cation in the apoplastic solution of the analyzed plant species was potassium, representing about 70–80% of the total cation concentration when the main anions were nitrate and chloride. In *Spinacia oleracea*, oxalate was also an important anion, while apoplast from *Hordeum vulgare* contained high amounts of malate. The charge balance was equal and total contents of cations and anions were between 10 and 20 mM, respectively. The activity of plasma membrane-bound H^+^-ATPase and membrane transport of solutes determine the pH condition of apoplast but pectic substances in the cell walls also affect the ion concentrations and pH in apoplast. The measured pH range of apoplast by pH electrode in apoplast of different plants varies from 4.5 to 7 (Sakurai, 1998).

The plant cell wall is mainly composed of polysaccharides such as celluloses, hemicelluloses and pectins, which interlock to form a dense and complex network. Additional compounds such as lignins, waxes or cutins are synthesized to form the secondary wall in specific differentiated cells (Carpita and Gibeaut, 1993). The cell wall acts as a passive barrier limiting the access of pathogens to plant cells and in turn, pathogens, especially necrotrophs, actively synthesize cell wall-degrading enzymes to penetrate or kill the plant cell (Laluk and Mengiste, 2010). The plant cell wall is actively remodeled and reinforced specifically at discrete sites of interaction with pathogens (Hamann, 2012; Underwood, 2012). This active reinforcement through the deposition of cell wall appositions, referred to as papillae, is one of the earliest responses to pathogen attacks (Micali et al., 2011). However, the cell wall proteome under a biotic stress is still poorly characterized. Identifying the proteins present in the apoplast during a pathogen infection is therefore essential to understanding the perception and regulation processes occurring between the two protagonists. A proteomic analysis that provides an overview of the protein pool at a given time is thereby an appropriate tool to address this issue and identify the actors involved in the interaction. Through this review, we will begin by pointing out the technical constraints to recovering apoplastic samples for proteomic analyses. We will then highlight the major findings obtained from the apoplastic proteome patterns during plant-pathogen interactions. In perspective, we will suggest future approaches to characterize early protein interconnections taking place between the pathogen and its host in the apoplast.

## Secretome and apoplastic proteome isolation

Despite the importance of apoplastic proteome during a given plant-pathogen interaction, it remains poorly characterized compared to the intracellular proteome. This is especially due to the difficulty in obtaining sufficient apoplastic material without damaging the plant cell and in avoiding potential contamination of the sample with cytoplasmic proteins. The proteins secreted in the apoplast are either soluble in the apoplastic fluid or ionically bonded to the plant cell wall (Soares et al., 2007). The literature uses different terms for the extracellular proteome present in the apoplast, the most common being “secretome” and plant “cell wall proteome.” For clarity and convenience, in this review, we will use the term “secretome” to designate secreted proteome obtained from *in vitro* cell suspension cultures, “apoplastic proteome” for soluble proteins present in the apoplastic fluid (generally extracted by the VIC method discussed below) and “cell wall proteome” for the secreted proteins that are loosely ionically bonded to the cell wall. The cell wall proteome is generally obtained from purified cell walls produced by disruptive methods. The cell wall proteome has been well investigated and excellent reviews have been published recently (Jamet et al., 2008; Rose and Lee, 2010; Albenne et al., 2013; Komatsu and Yanagawa, 2013).

Several studies have used suspension cell cultures such as *in vitro*-simplified models to develop practical, simple and non-destructive methods to isolate secreted proteins. The suspension cell cultures are easy to maintain, handle and scale up/down and the secreted proteins in the culture medium can be easily separated from suspended cells by filtration without cell disruption. Therefore, this system facilitates the extraction of freely soluble secreted proteins by the plant cells in suspension cultures and greatly limits potential contamination by cytoplasmic proteins. This simplified approach has been used to characterize basal secretomes of different species like *Arabidopsis* (Oh et al., 2005), alfalfa (Kusumawati et al., 2008), tobacco (Okushima et al., 2000), or rice (Cho et al., 2009). These *in vitro* systems have been used to assess cell responses more easily following signal molecules or fungal-derived elicitor treatments (Table 1). The effect of the well-known signal molecules SA and JA was characterized on *Arabidopsis* and grapevine cell suspension secretomes, respectively (Oh et al., 2005; Cheng et al., 2009; Martinez-Esteso et al., 2009). Comparative secretome studies have been performed with chitosan and *Fusarium*-based elicitors on *Arabidopsis* and maize (Ndimba et al., 2003; Chivasa et al., 2005). These studies revealed proteome changes in response to individual MAMPs but without taking into account the complexity of the responses triggered during a typical plant-microorganism interaction. To our knowledge, only two studies have directly used the pathogen itself, demonstrating that the study of plant-pathogen interactions has proven to be very difficult in these *in vitro* systems (Kaffarnik et al., 2009; Kim et al., 2009). Even then, the *in vitro* secretome analysis only provides partial identification of the secreted proteins in comparison to the *in planta* apoplastic proteome analysis as demonstrated by the comparative analysis of *in vitro* secretome and leaf apoplastic proteomes in rice (Jung et al., 2008). Analyses of whole secreted proteins identified 222 protein spots with only 6 protein spots common to both *in planta* and *in vitro* samples. The proteins involved in cell wall metabolism in relation with plant defense mechanisms represent 18% of the total proteins identified *in planta* compared to 64% *in vitro*.

**Table 1:** **Main secretome studies on *in vitro* plant cell suspension under an elicitor or pathogen treatment**.

**Plant**	**Proteome type**	**Biotic treatment**	**Sample preparation**	**Analysis method**	**Key findings**	**References**
*Arabidopsis thaliana*	S; CWP	Chitosan and Fusarium-based elicitor	Filtration and acetone precipitation	2D-PAGE and MALDI-MS or ESI-MS/MS	2 glucanases, 1 peroxidase, 1 chitinase, 1 polyglacturonase, 1 carboxypeptidase, and 1 receptor-like kinase differentially expressed in S or CWP. Potential extracellular phosphorylation of glucanase, chitinase and receptor-like kinase	Ndimba et al., 2003
*Arabidopsis thaliana*	S	SA 0.5 mM	Membrane filtration under vacuum lyophilisation and dialysis	2D-PAGE and MALDI-TOF/MS	13 differentially expressed proteins identified. Identification and characterization of GDSL motif lipase (GLIP1) involved in *A. brassissicola* resistance trough ethylene pathway	Oh et al., 2005
*Arabidopsis thaliana*	S	SA 0.5 mM and SA 1 mM	Membrane filtration, lyophilisation, dialysis, acetone precipitation	2D-PAGE and LC-MS and Q-TOF-MS	63 differentially expressed proteins within 2 h after SA treatment, mainly involved in metabolism (34%), defense (13%) or binding function (12%)	Cheng et al., 2009
*Arabidopsis thaliana*	S	*Pseudomonas syringae* p.v. Tomato strains: DC3000 (virulent), AvrRpm1 (avirulent) and HrpA (non-pathogenic)	Filtration and phenol extraction acetone precipitation	iTRAQ combined with LC-MS/MS and Q-TOF-MS	45 differentially expressed proteins identified, mainly involved in metabolism (18%), redox regulation (18%), defense (11%), or cell wall maintenance (26%). DC3000 and AvrRpm1 strains induce proteins without secretion peptide signal	Kaffarnik et al., 2009
*Zea mays*	S; CWP	Chitosan, H_2_O_2_ and Fusarium-based elicitor	S: filtration and acetone precipitation CWP: Cell-wall fragment isolation, CaCl_2_/urea extraction and acetone precipitation	2D-PAGE and MALDI-TOF/MS and nanoHPLC-MS/MS	Glucosaminidase, glyceraldehyde-3P-dehydrogenase, peroxidase, and xylanase inhibitor are treatment-dependent regulated through probable dephosphorylation	Chivasa et al., 2005
*Vitis vinifera* (cv Gamay)	S	Methylated cyclodextrins 50 mM, and/or methyl jasmonate 0.1 mM	Centrifugation, ethylacetate extraction and TCA precipitation	2D-PAGE and MALDI-TOF/MS or LC-MS/MS	25 differentially expressed protein spots lead to identification of 10 proteins: peroxidases, chitinase, glucanase, thaumatin-like, lipase-like, PR27, endotransglycosylase, subtilisin-like protease	Martinez-Esteso et al., 2009
*Oryza sativa*	S	*Magnaporthe grisea* and its derived elicitor	Vacuum filtration, phenol-methanol-ammonium extraction and acetone wash	2D-PAGE and MALDI-TOF/MS or μ LC-ESI-MS/MS	21 differentially expressed proteins identified with mainly defense-related functions (chitinases, oxalate oxidase) and domain unknown function 26 proteins (DUF26). Stronger and earlier induction of transcripts *in planta* with virulent strains	Kim et al., 2009

*S, secretome; CWP, cell-wall proteome*.

Since the *in vitro* approach does not provide a natural environment for the cells and because physiologically relevant treatments are difficult to apply, recent studies were instead carried out *in planta*. In this case, when a plant organ such as a leaf is required for study, apoplastic fluid is most commonly isolated using the vacuum infiltration centrifugation (VIC) method, well described by Lohaus et al. (2001) and Agrawal et al. (2010). In short, the plant leaves are thoroughly rinsed with a buffer to reduce the leaf surface tension and facilitate the vacuum infiltration. After infiltration with the adapted extraction buffer, the leaf surfaces are quickly dried to avoid sample dilution and the carefully rolled leaves are transferred to 50 ml polypropylene tubes with a washer at the bottom to avoid immersion of leaves into the collected apoplastic fluid. The gentle centrifugation allows the recovery of the apoplastic fluid from which apoplastic proteins are extracted by precipitation. The VIC method has been used to characterize the apoplastic proteome of different plant species such as *Arabidopsis* (Casasoli et al., 2008), rice (Cho et al., 2009), tobacco (Delannoy et al., 2008), maize (Witzel et al., 2011), alfalfa (Soares et al., 2007), pea (Wen et al., 2007), tomato (Konozy et al., 2012), or grapevine (Delaunois et al., 2013). This VIC method has also been used to assess the apoplastic proteome changes occurring after an elicitor, a pathogenic or non-pathogenic treatment (Table 2). Casasoli et al. (2008) have used oligogalacturonides on *Arabidopsis* to identify apoplastic candidate proteins involved in the response to this elicitor and the proteins associated with H_2_O_2_ response was investigated in the rice root apoplast (Zhou et al., 2011). Apoplastic proteome changes were studied during plant interactions with pathogenic bacteria (*Agrobacterium tumefaciens* and *Pseudomonas syringae*) and fungal pathogens, mainly *Verticillium longisporum* and *Magnaporthae oryzae* (Table 2). However, methodological adaptations might be necessary for efficient sample preparation. For instance, a water-displacement method was developed to obtain apoplast fluids from stem tissues in the poplar/*Melampsora medusa* interaction (Pechanova et al., 2010). The VIC technique allows the apoplastic proteome extraction without much cell damage. However, at times, the fragility of the samples leads to the rupture of the cytoplasmic membrane, triggering varying contamination by cytoplasmic proteins. The apoplastic fluid sample requires more stringent assessment of intracellular contamination to ensure apoplastic fraction purity. To estimate cytoplasmic contamination, enzyme activity, immunoblotting or electrolyte leakage can be used. Malate dehydrogenase activity is the most commonly used measure to estimate the level of membrane damage caused by the VIC method. Antibodies directed against malate dehydrogenase, RuBisCo or ATPase are also frequently used to determine the contamination level (Delaunois et al., 2013). Electrolyte leakage and concentration of malondialdehyde, which is a breakdown product of membrane lipid peroxidation, can also be used (Zhou et al., 2011).

**Table 2:** **Main apoplastic proteome studies after an elicitor or pathogen treatment**.

**Plant**	**Proteome type**	**Biotic treatment**	**Sample preparation**	**Analysis method**	**Key findings**	**References**
*Arabidopsis thaliana*	AP	Oligogalacturonides (100 mg/mL)	Vacuum infiltration centrifugation and TCA precipitation	2D-DIGE and MALDI-TOF/MS or LC-MS/MS	16 differentially expressed proteins identified like polygalacturonase inhibitor, α-glucosidase, LRR protein, DUF26 receptor like Identified proteins putatively involved in pathogen perception and protein PTM regulated by OGs	Casasoli et al., 2008
*Arabidopsis thaliana*	AP	*Verticillium longisporum*	Vacuum infiltration centrifugation and TCA precipitation	2D-PAGE and ESI-LC-MS/MS	Specific increase in 6 proteins (3 peroxidases, 1 serine carboxypeptidase, 1 α-galactosidase, and 1 germin-like protein GLP3). Main function in carbohydrates modifications (25%), oxidoreductions (21%) proteases (18%), defense, and cell wall modification	Floerl et al., 2012
*Arabidopsis thaliana* and *Medicago sativa*	REP	*Pseudomonas syringae* p.v. Tomato DC3000 and *Sinorhyzobium meliloti* Rm1021	Filtration, concentration through ultracentrifugal filter and TCA precipitation	2D-PAGE and nanoHPLC-MS/MS	More than 100 identified proteins are differentially accumulated during plant microbe interaction.Seven plant proteins and four bacterial proteins increased during *S. meliloti/alfalfa* interaction and nine plant defense-related proteins increased during *P.syringae* DC3000/Arabidopsis compatible interaction	De-La-Pena et al., 2008
*Brassica napus*	AP	*Verticillium longisporum* spores on roots	Vacuum infiltration centrifugation and TCA precipitation	2D-PAGE and ESI-LC-MS/MS	Four differentially expressed proteins identified: basic glucanase, β-1,3-glucanase, basic endochitinase and peroxidase	Floerl et al., 2008
*Populus deltoides*	AP	*Melampsora medusae* and *Melampsora larici-populina*	Fluid exudation under pressure and phenol-methanol-ammonium extraction and acetone wash	2D-PAGE MALDITOF-MS/MS and 2D-LC ESI-MS/MS	Leaf and stem apoplast proteomes were analyzed with mainly stress/defence related proteins, cell wall metabolism, and antioxidative function (chitinases, glucanases, peroxidase, and antioxydant enzymes are the more represented)	Pechanova et al., 2010
*Nicotiana benthamiana*	AP	*Agrobacterium tumefaciens*	Vacuum infiltration centrifugation and chloroforme-methanol precipitation	2D-PAGE and LC-MS/MS	PR proteins increased greatly upon infection representing 45% of the spot volume and cell wall-modifying enzymes represents 15% of the total protein content	Goulet et al., 2010
*Oryza sativa*	AP	0.3 and 0.6 mM of hydrogen peroxide (H_2_O_2_)	Vacuum infiltration centrifugation, filtration, centrifugal concentration and TCA precipitation	2D-PAGE and MALDI-TOF/TOF and MS/MS	35 differentially expressed proteins identified, with around half related to redox state regulation and other involved in cell wall modification, signal transduction, cell defence, and carbohydrate metabolism	Zhou et al., 2011
*Oryza sativa* ssp. Japonica cv Kakehashi	AP	*Magnaporthe oryzae* Ken54-20 (incompatible) or Ina186-137 (compatible)	Vacuum infiltration centrifugation and TCA precipitation	2D-PAGE and ESI-LC-MS/MS	Three differentially expressed domain unknown function 26 proteins (DUF26) identified at 12 hpi and 5 defense-related proteins at 72 hpi	Shenton et al., 2012
*Oryza sativa* ssp. Japonica cv Jinheung	AP	*Magnaporthe oryzae* KJ401 (incompatible) or KJ301 (compatible)	Vacuum infiltration centrifugation and phenol-methanol-ammonium extraction and acetone wash	2D-PAGE/MudPIT and MALDI-TOF/MS or nESI-LC-MS/MS	More than 730 identified proteins with 40% and 60% derived from rice and *M oryzae*, respectively, mainly stress and ROS-related function for rice and metabolism and cell wall hydrolysis for *M oryzae*. Differential expression under compatible/incompatible interaction was confirmed by RT-PCR	Kim et al., 2013

*AP, apoplastic proteome; REP, root exudates proteome*.

## Protein separation, identification, and quantification techniques

The main problem with the VIC technique is the extremely low yield implying that either very large volume of samples have to be produced and concentrated or very sensitive methods have to be used for proteomic analysis. A significant amount of information has been gained from proteomic studies using classical gel-based separation, as resolved proteins can often be identified and further characterized by mass spectrometry (MS). Two-dimensional polyacrylamide gel electrophoresis (2D-PAGE) involves resolving proteins by isoelectric point (p*I*) and molecular weight (Görg et al., 2004; Kav et al., 2007). The 2D-PAGE has mainly been used for identifying plant protein abundance alterations in secretome or apoplastic proteomes in response to biotic stresses and still remains a viable technique (Shenton et al., 2012). Fluorescence difference gel electrophoresis (DIGE) was developed as a more quantitative form of 2D-PAGE. Here, samples are differentially covalently labeled with fluorophores, allowing for distinction between proteins resolved on the same gel (Casasoli et al., 2008). Casasoli et al. (2008) used this technique in *Arabidopsis* to identify the differential expression of 62 proteins in the same gel between control and oligogalaturonide-treated apoplastic proteomes. Both 2D-PAGE and DIGE approaches control for gel-to-gel variation, but do not overcome the issues of spot overlap (Campostrini et al., 2005). The gel-free methods utilizing liquid chromatography (LC) techniques for separating peptides after sequence-specific digestion can overcome this issue and significantly increase proteome coverage (Roe and Griffin, 2006). For example, multidimensional protein identification technology (MudPIT), which combines strong cation exchange with reversed phase chromatography, has been used on rice leaves infected with the fungus *Magnaporthe oryzae* (Kim et al., 2013). During this interaction, over 730 secretory proteins were identified in the apoplast by combining 2D-PAGE and MudPIT techniques, 40% and 60% of these corresponding to rice and *Magnaporthe oryzae*, respectively. Increasingly, gel-based, and gel-free separation methods are used together as complementary techniques to increase the number of identified proteins (De-La-Pena et al., 2008; Cheng et al., 2009; Pechanova et al., 2010; Kim et al., 2013).

Protein identification is performed by mass spectrometry (MS) analysis. The three main steps are protein or peptide ionization, ion separation and detection. Matrix assisted laser desorption/ionization (MALDI) and electrospray ionization (ESI) are the two main ionization techniques that are applied in apoplastic proteome studies while ion separation and detection uses mainly time-of-flight (TOF) or quadrupole mass analyzer. Tandem MS (MS/MS) is now commonly used to improve the sensitivity and accuracy of peptides/proteins identification and the different techniques are very often combined (like MALDI-TOF/MS or ESI-MS/MS, Tables 1, 2). The identification of proteins present in the apoplast in a given plant-pathogen interaction implies an access to a proteome and/or a genome database of the two organisms, which is not always the case. Alexandersson et al. (2013) suggest the use of a combined plant-pathogen protein database extended with a random sequence database to avoid false positive hits from host peptides when matching pathogen peptides.

Plant-pathogen interaction mechanisms involve the fine modulation of protein amount. Precise and sensitive quantification methods of proteins become essential. Staining on polyacrylamide gels with Coomassie blue or silver nitrate is generally performed for spot quantification. However, quantifications on stained spots are difficult to interpret for several reasons: overlapping spots can occur, different proteins can be present in the same spot or some proteins can be present in different spots due to post-translational modifications (PTM), and degradation or maturation of proteins. The labeling of proteins with fluorescent dyes prior to electrophoresis (DIGE) can partially overcome some of these issues (Casasoli et al., 2008). More recently, protein quantification was significantly improved in proteomics using *in vitro* chemical (ICAT or iTRAQ) or *in vivo* metabolic (SILAC or ^15^N-labeling) isotope-assisted quantification methods. For *in vivo* metabolic stable isotope labeling, cell suspension cultures or plants are grown on media supplemented with heavy isotope-containing amino acids or ^15^N-labelled nutrients, allowing for the labeling of proteins as they are synthesized (Jayaraman et al., 2012). However, this approach is not always easy to implement *in planta* and requires long and powerful bioinformatics analysis. Kaffarnik et al. (2009) analyzed the secretome of *Arabidopsis* in response to infection by *Pseudomonas syringae* using a recently developed technique known as isobaric tag for relative and absolute quantification (iTRAQ). In this method, labeling is chemically performed *in vitro* on amines of digested peptide samples with commercially available iTRAQ (isobaric tags) reagents. The major advantage is that this strategy can be applied to directly compare up to eight separate samples in one experiment. With this technique Kaffarnik et al. (2009) compared apoplastic proteomes of *Arabidopsis* infected with three different strains of *Pseudomonas syringae* p.v. tomato, strain DC3000 (virulent), strain DC3000 carrying AvrRpm1 (avirulent) and strain DC3000 knocked-out for HrpA (non-pathogenic), suggesting a pathogen-mediated manipulation of apoplastic proteins. The development and the more systematic application of these isotope-assisted quantification and gel-free methods should allow the identification of low-abundance apoplastic proteins or small variations in their level of expression in the near future.

## Leaderless secretion proteins

Plant proteins are secreted to the apoplast mainly via the classical ER-Golgi route. SignalP or TargetP software is widely used to predict signal peptides from the sequences (Emanuelsson et al., 2007; Petersen et al., 2011) and to identify proteins that are secreted through the classical ER-Golgi. However, there is increasing evidence that a subset of apoplastic proteins is likely to be secreted by non-classical pathways. Non-classical or leaderless secretion is common to all eukaryotes, including plants. Computational analysis using different algorithms have been developed to assist in the identification of unexpected secreted proteins. SecretomeP allows secretion prediction based on sequence features conserved or frequently observed in secreted bacterial and mammalian proteins (Bendtsen et al., 2004). Cheng et al. (2009) found that 60% of the Leaderless Secreted Proteins (LSPs) identified in the *Arabidopsis* secretome were predicted to be secreted with SecretomeP. Among these predicted LSPs actually found in the apoplast, we can mention calmodulin, jacalin, or superoxide dismutase. Although no identified plant superoxide dismutase has a signal peptide, extracellular superoxide dismutase activity in stressed or pathogen-infected plants has been previously reported (Hernández et al., 2001; Karpinska et al., 2001). In the same way, calmodulin is known to be an intracellular calcium sensor, but it has recently been suggested that calmodulin could serve as a dual messenger with roles either inside or outside the cell depending on stress factors (Cui et al., 2005). However, it should be noted that SecretomeP software may not be well-adapted to plant proteins since it has been designed for mammalian proteins. In addition, only a small proportion of the LSPs identified in apoplastic proteome studies gave a score above threshold (Cheng et al., 2009; Fernandez et al., 2012). The difficulties in preserving membrane integrity and extracting non-contaminated apoplastic fluids combined with the limitations of the bioinformatics programs in predicting sub-cellular localization have to be taken into account to understand the contrasting variations of LSPs content between experiments. New computational tools such as software or databases are emerging and should help to predict more precisely and with greater certainty the proteins produced through alternative secretion pathways. LocTree3 is a new software program that predicts protein subcellular localization through a consistent new framework with a high prediction success especially for secreted proteins (https://rostlab.org/services/loctree3) (Goldberg et al., 2012). The comparative platform OrysPSSP is composed of a core “small secreted protein” (SSP) database and a dynamic web interface that integrates a variety of user tools and resources and allows the screening of SSP on the genome scale and across the phylogeny of plant species (http://www.genoportal.org/PSSP/index.do) (Pan et al., 2013).

The existence of these alternative secretory routes could be explained by the need of rapid and effective regulation of secretion to provide a selective advantage to the plant cell. There is growing evidence of complex and highly coordinated spatiotemporal protein secretion in plants. Kaffarnik et al. (2009) showed that most of the proteins induced in *Arabidopsis* by the virulent *Pseudomonas syringae* DC3000 or the avirulent AvrRpm1 strains had no secretion signals as compared to non-pathogenic HrpA strains. Cheng et al. (2009) showed in *Arabidopsis* cell-suspensions treated with SA that 65%, 50%, and 35% of the secreted proteins lack a peptide signal after 1, 2, and 6 h, respectively. These results suggest that external stresses rapidly induce enhanced protein secretion. Other explanations could be the accumulation of inactive pre-proteins prior elicitation or posttranslational modifications made by the ER environment, which may not be required for specific activity of apoplastic proteins (Rose and Lee, 2010).

## Post-translational modifications

Proteomic studies lead very often to the identification of the same protein in different spots suggesting different post-translational modifications (PTMs) of the same protein (Chivasa et al., 2005). Indeed, PTMs are known to control many physiological processes by affecting protein structure, activity, and stability. Proteins can undergo different PTMs such as glycosylation, phosphorylation, carbonylation, or nitrosylation (Jayaraman et al., 2012; Albenne et al., 2013). The secretome analysis of *Arabidopsis* cell suspensions in response to oligogalacturonides highlighted several protein isoforms, such as an alpha xylosidase and a receptor-like kinase, showing differential PTMs (Casasoli et al., 2008). This observation may suggest a role for PTMs in the plant response to pathogens. Glycosylation is one of the most common and complex PTM. There are two main types of glycosylation, namely *N*- and *O*-glycosylation, but plant glycoproteins still remain poorly characterized. Glycoproteomics are currently applied to plants (Albenne et al., 2013). ConA lectin chromatography approaches were used to specifically isolate *N*-glycoproteins from *Arabidopsis* (Minic et al., 2007) and tomato (Català et al., 2011). The use of a multi-dimensional lectin chromatography system increased the coverage of the *Arabidopsis* cell wall glycoproteome leading mainly to the identification of *N*-glycosylated proteins (Zhang et al., 2011). The regulation of enzymes putatively involved in glycosylation, such as the disappearance of a β − *N* − acetylglucosaminidase in the elicitor-treated maize secretome (Chivasa et al., 2005), suggests that glycosylation might also occur in the apoplast. The modulation of post-translational glycosylation would quickly regulate the activity and/or structure of targeted proteins, potentially strengthening the cell wall through stronger cross-linking.

Phosphorylation plays a key role in signal transduction and is based on the reversible regulation of the transfer of a phosphoryl group bonded to an aminoacid by protein kinases or removed by phosphatases. Although different phosphospecific staining techniques were developed for phosphoproteomic studies, LC-MS/MS analysis following gel-free separation and phosphopeptide enrichment is often the method of choice (Grimsrud et al., 2010). Upon perception of microbial signals, kinases and phosphatases target specific proteins, often modifying complex signaling cascades that allow for rapid defense responses. Ndimba et al. (2003) have shown that chitosan treatment of *Arabidopsis* cell-suspensions induce phosphorylation of a receptor-like kinase, endochitinases and glucanases. Similarly, Chivasa et al. (2005) have positively identified in the maize secretome phosphotyrosine protein spots that are rapidly dephosphorylated in response to *Fusarium*-elicitor treatment. The presence of elicitor-induced changes in the phosphorylation status of extracellular proteins suggests the existence of pathogen-induced, phosphorylation/dephosphorylation-regulated intercellular signaling via the extracellular matrix. Moreover the presence of phosphatases in the extracellular proteome of *Arabidopsis* infected by *Pseudomonas syringae* suggests that potential phosphorylation/dephosphorylation reversible regulation could occur in the apoplast (Kaffarnik et al., 2009). A recent comparison of *Lotus japonicus* roots elicited with symbiotic-(Nod factors) and the MAMP flg22 revealed differential phosphorylated protein patterns between symbiotic and defense responses (Serna-Sanz et al., 2011).

Carbonylation is considered as a marker of protein oxidation, which results from the direct oxidation of various aminoacids. This PTM is involved in the control of the protein function and can lead to their degradation (Lounifi et al., 2013). The early oxidative burst in response to pathogen attacks is leading to an increase of protein carbonylation (Zhang et al., 2007). Although there is a strong link between ROS and pathogen attack on one side and the ROS and protein carbonylation on the other side, so far no large-scale study has been conducted on the regulation of protein carbonylation during a given plant-pathogen interaction. As mentioned before, analysis of the apoplastic proteome under biotic stress has revealed an important part of the proteome changes involved in the ROS metabolism. This correlation suggests that large changes in ROS metabolism-related proteome in the apoplast would influence the redox balance and consequently the protein carbonylation level. The resulting rapid PTM activates other defense-related proteins.

Protein nitrosylation is considered as one of the key mechanism regulating protein function (Lounifi et al., 2013). Since nitrosylation refers to the covalent bonding of a NO molecule to the cysteine amninoacid it becomes apparent that NO species produced upon plant pathogen interactions can exert their signaling action through nitrosylation of specific proteins (Corpas et al., 2008; Spoel and Loake, 2011). A large-scale proteomic study conducted on *Arabidopsis* leaves treated with gaseous NO led to the identification of 25 nitrosylated proteins which are involved in stress response, redox status, signaling, and cytoskeleton functional categories. *Pseudomonas syringae* infection of *Arabidopsis* seedlings leads to a hypersensitive response accompanied by an NO burst and triggers an increase of nitrosylated proteins. Most of the identified nitrosylated proteins were enzymes involved in intermediary metabolism, signaling, and antioxidant defenses (Romero-Puertas et al., 2008). Therefore the extent of protein nitrosylation could be expected to change in response to NO-originated stimuli governed by pathogen infection. Moreover, the occurrence of a biological connection between protein oxidation and nitrosylation in plants appears to be increasingly documented (Lin et al., 2012). Since the ROS-based protein carbonylation and the NO-based protein nitrosylation, as well as their interactions, seem to act as major regulatory systems in stress responses, the characterization of protein oxidation and nitrosylation in plant-pathogen interactions becomes crucial to the understanding of the various physiological processes occurring in the apoplast. Plant PTM proteomics is still in its early stages and is undoubtedly a promising approach to gain new insights into the structure and function of apoplastic proteins during pathogen infection.

## Main findings from apoplastic proteomics case studies

### Perception and signal transduction

**Figure 1:**
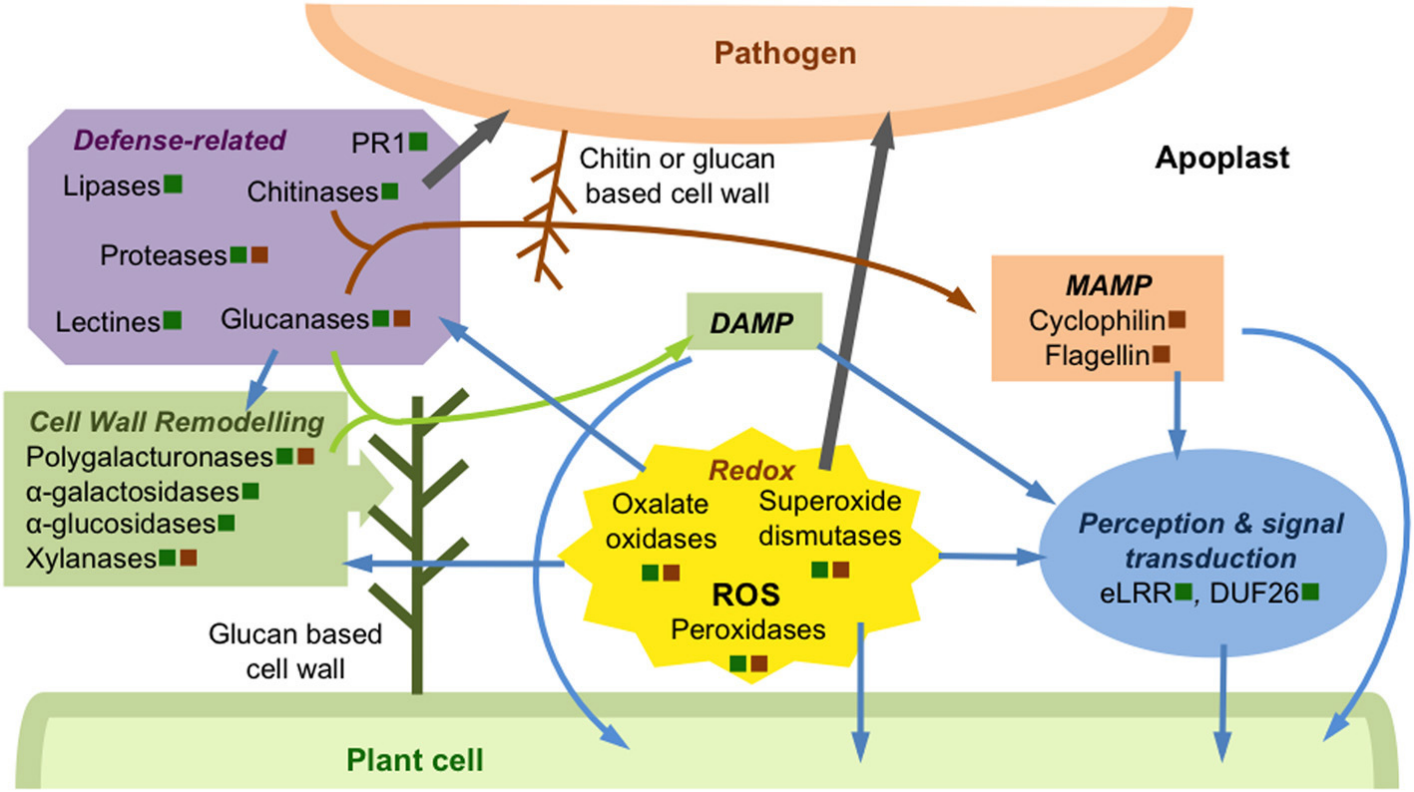
**Schematic overview of some events occurring in apoplast during plant pathogen interactions**. This illustration, based on studies described in this paper, presents some examples of apoplastic proteins regulated during biotic stresses. These proteins are secreted by the plant cell (green square) and/or pathogen (brown square). Some proteins, modulated in the apoplast after DAMP or MAMP perception, are involved in pathogen perception and signal transduction, leading to the activation of intracellular plant defense signaling pathways (blue arrows). The regulation of a large amount of proteins involved in redox homeostasis modulates the ROS signaling pathway leading to activation of extra- and intracellular plant defense responses (blue arrows). These proteins, such as peroxidases or oxalate oxidases participate also to the plant cell defense through plant cell wall reinforcement or direct pathogen attack (gray arrow). Plant cell wall is actively remodeled and/or reinforced through the regulation of numerous enzymes, such as polyglacturonases or glucanases, which are secreted by the pathogen or its host. Some of the cell wall degradation products can act as DAMP (green arrow) to stimulate plant defense signaling pathways. Most of the defense-related proteins, such as chitinases or glucanases, act directly against the pathogen (gray arrow), releasing degradation products that can be perceived as MAMP by the plant cell (brown arrow).

As mentioned before, early perception of the pathogen occurs in the apoplastic compartment and several proteomic studies have highlighted the regulation of apoplastic proteins potentially involved in pathogen perception and signal transduction. These apoplastic proteins generally feature LRR-type motifs suggesting a potential receptor role for these proteins and they sometimes undergo PTM like phosphorylation suggesting an involvement in signal transduction cascades. In the secretome of *Arabidopsis* suspension cultures elicited with chitosan, the phosphorylation of a cell wall bound putative receptor-like protein suggests that elicitor-treatment involves signal transduction cascades initiated in the apoplast through accumulation of phosphorylated extracellular receptor-like proteins (Ndimba et al., 2003). In the apoplast of *Arabidopsis* leaves elicited by oligogalacturonides, Casasoli et al. (2008) observed the accumulation of a disease resistance related LRR protein, characterized by a LRR domain composed of 13 repeats of the extracytoplasmic type (eLRRs), and previously localized in the plant cell wall (Borderies et al., 2003; Bayer et al., 2006) (Figure 1). The correlation of this LRR protein accumulation with the induction of its corresponding transcript as well as the reported induction of its gene during the incompatible interaction with *Alternaria brassicicola* suggest a role in pathogen perception (Schenk et al., 2003).

Receptor-like kinases with DUF26 domains are another class of interesting proteins related to biotic stress, regulated at both the protein and the transcript level, but with still unknown functions. DUF26 genes were previously found to be up-regulated upon pathogen infection, JA treatment (Kim et al., 2003, 2004b) and wounding (Shen et al., 2003). Recent studies have identified DUF26 proteins regulated upon pathogen infection or elicitor treatment (Kim et al., 2009, 2013; Zhou et al., 2011; Shenton et al., 2012). Several lines of evidence support the fact that these proteins could be involved in the perception of the pathogen or the transduction of the signal after perception. The proteins containing DUF26 domains, which are usually found in serine/threonine kinases, are annotated as small secretory proteins and are associated with plant receptor protein kinase domains in databases. These proteins accumulate earlier in incompatible rice/*Magnaporthe grisae* interactions than in compatible ones (Kim et al., 2009). Their extremely rapid accumulation was also demonstrated in the rice apoplast 12 h after infection with *Magnaporthe oryzea* (Shenton et al., 2012; Kim et al., 2013). Time-course profiling at the transcript level confirmed that their inductions were stronger and earlier in incompatible interactions.

Fine study of the apoplastic proteome during the early phase of the pathogen infection may also lead to the identification of proteins specifically produced by the pathogen and involved in the perception. For instance, a flagellin B homolog from *Agrobacterium tumefaciens* was shown to accumulate in the apoplast of tobacco agro-infiltrated leaves (Goulet et al., 2010) and the putative virulence factor cyclophilin CYP1 from *Magnaporthe* was found in the rice apoplast (Shenton et al., 2012). Similarly, De-La-Pena et al. (2008) showed evidence that bacteria are also able to change the proteins they secrete, depending on the identity of the plant partner. The elongation factor Tu was only secreted in the interaction between *Pseudomonas syringae* DC3000 and alfalfa but not with *Arabidopsis*, suggesting that stimulation of innate immunity by this bacterial protein could be plant-microbe dependent (De-La-Pena et al., 2008).

Kim et al. (2013) recently developed an interesting screening approach to identify putative apoplastic effectors secreted by *Magnaporthe oryzae* in rice leaves showing that some proteins activate the PBZ1 cell death related promoter only when they are expressed in the apoplast, acting putatively as apoplastic effectors. Therefore, apoplastic proteomic approaches may prove to be an interesting tool to discover or unravel key players involved in pathogen perception and signal transduction.

### ROS and redox regulation

Secretome or apoplastic proteome studies under biotic stress conditions generally reveal changes of proteins involved in ROS metabolism, representing 10–30% of the secreted proteome (Tables 1, 2). Members of most of these protein families, such as peroxidases, oxalate oxidases or superoxide dismutases, are generally present in the unstressed leaf apoplast (Delaunois et al., 2013). Unsurprisingly, the H_2_O_2_ treatment of rice seedlings triggers protein changes in the apoplast, 30% of which are involved in redox homeostasis. These proteins are involved in adjusting redox plant cell status, either triggering defense reactions or overcoming the deleterious effects of oxidative stress (Figure 1). It is noteworthy that a number of redox-associated enzymes such as some peroxidases are repressed at protein levels, which might modulate the H_2_O_2_ concentration to an appropriate level. At the same time, peroxidase accumulation in apoplast has been observed during the *Brassica napus/Verticillium longisporum* interaction (Floerl et al., 2008), in the secretome of grapevine cell suspension treated with MeJA (Martinez-Esteso et al., 2009) or in the secretome of *Arabidopsis* cell suspension treated with SA (Cheng et al., 2009), revealing that either the pathogen or a related molecule signal is able to modulate the level of peroxidases in the apoplast. Moreover, the peroxidase accumulation was correlated with their transcript accumulation in the *Populus/Melampsora* interaction (Pechanova et al., 2010) and the *Arabidopsis/Verticillium* interaction (Floerl et al., 2012). However, the time-course and the degree of transcript accumulation could be different from the corresponding protein levels. Finally, in maize secretome, some peroxidases do not change in quantity but are dephosphorylated after *Fusarium*-elicitor treatment (Chivasa et al., 2005) suggesting a precise regulation of peroxidases in the apoplast itself through PTM. Plants respond to bacterial challenge through quantitative and qualitative changes in peroxidase secretion leading to symbiotic or defense responses. During the rice-*Magnaporthe oryzae* interaction, Kim et al. (2013) have observed the modulation of 20 different peroxidases suggesting an intracellular ROS homeostasis to maintain a delicate equilibrium. Similarly, the root exudate proteome analyzed during the interaction between alfalfa and the bacterial symbiont *Sinorhizobium meliloti* or between *Arabidopsis* plants and an opportunistic bacterial pathogen *Pseudomonas syringae* revealed a complex and fine-tuned regulation of peroxidase amount depending on the plant-bacterium combinations (De-La-Pena et al., 2008). The existence of large multigenic families of peroxidases in plants (with 138 members in rice and 73 members in *Arabidopsis*) with a high number of enzymatic isoforms warrants their complex and fine-tuned regulation. Peroxidases were especially involved in a broad range of plant defense mechanisms such as lignin and suberin formation, cross-linking of cell wall components, phytoalexin synthesis, and the metabolism of ROS and RNS (Almagro et al., 2009). It should be noted that during most biotic stress responses, the major sources of ROS seem to be due to cell wall localized peroxidases that generate hydrogen peroxide, or plasma membrane-localized NADPH/NADH oxidases that generate superoxide, or both systems operating in tandem (Bolwell, 1999; Daudi et al., 2012). Since the NADPH oxidases are plasma membrane localized (Lherminier et al., 2009), unsurprisingly, they are not found in the apoplast of pathogen infected plants, whereas the amount of numerous peroxidases is finely modulated. The absence of these proteins in the apoplastic fluid could even be used as a marker of the plasma membrane integrity as well as H^+^-ATPases.

Among all the proteins identified in the apoplastic proteome and involved in the regulation of ROS, we can also mention two oxalate oxidases (or germins) that highly accumulate in rice suspension-cultured cells treated with a rice blast fungus elicitor (Kim et al., 2009). The oxalate oxidases are involved in responses to biotic or abiotic stresses by producing H_2_O_2_ from oxalic acid. Since certain fungal pathogens produce oxalic acid, the oxalate oxidases present in apoplast could degrade the oxalic acid produced upon fungal infection to generate H_2_O_2_, which in turn may functions as a signal for plant defenses (Figure 1).

The antioxidant enzymes superoxide dismutases are also involved in ROS signaling and significantly accumulate in the secretome of *Arabidopsis* cell suspensions treated with SA (Cheng et al., 2009) or inoculated with *Pseudomonas syringae* (Kaffarnik et al., 2009). They also accumulated in the apoplastic poplar proteome infected by *Melampsora medusae* (Pechanova et al., 2010). Superoxide dismutase produce H_2_O_2_ from superoxides (O^·−^_2_), which is further degraded to H_2_O by ascorbate peroxidase. The superoxide dismutase could favor the transient nature of the oxidative burst and prevent accumulation of toxic superoxides, limiting the duration of the oxidative burst to an early event in plant defense (Scheler et al., 2013). Similarly, *Sinorhizobium meliloti* secretes higher amounts of superoxide dismutase in alfalfa than *Arabidopsis* roots, suggesting that the bacteria specifically recognize alfalfa to initiate the symbiosis (De-La-Pena et al., 2008).

Overall, these proteomic approaches reveal a strong implication of the apoplastic proteins involved in ROS homeostasis. They also highlight the fine regulation of these proteins requiring the control of their secretion as well as their activation through post-translational modifications depending on the plant-pathogen interaction.

### Cell wall modification

As mentioned before, the cell wall is one of the most important barriers to counter pathogen invasion and it is not surprising to find in the unstressed apoplast numerous enzymes involved in cell wall modification or maintenance (Albenne et al., 2013; Komatsu and Yanagawa, 2013). It has already been described how the cell wall is actively remodeled and reinforced during infection specifically at discrete sites of interaction with pathogens (Figure 1) (Hamann, 2012; Underwood, 2012). However, a global view of the regulation of the enzymes specifically involved in the cell wall remodeling following a pathogen invasion is still incomplete. Apoplastic proteomic approaches definitely help to decipher the regulation of this complex metabolism involving numerous enzymes. Indeed, in most of the studies listed in Tables 1, 2, biotic stresses modulate the accumulation of secreted proteins involved in cell wall modification or maintenance like polygalacturonases, α-galactosidases, α-glucosidases, xylanases, xyloglucanases, and β-1,3-endoglucanases. Peroxidases (see above) also participate in cell wall reinforcement by modifying carbohydrate and structural protein polymer networks (Albenne et al., 2013) or through lignification or suberisation (Ndimba et al., 2003). Enzymes that breach the plant cell wall have also been shown to be important for fungal pathogens that lack specialized penetration structures and for necrotrophic pathogens. For example, the polygalacturonases are among the most extensively studied enzymes. They cleave the linkages between α-1,4 D-galacturonic acid residues, which are the major component of pectin, to produce non-methylated homogalacturonan. Polygalacturonases cause cell separation, tissue maceration, and release of mono- di- and three- saccharides used as nutrients by the pathogen (De Lorenzo et al., 2001). Some of these released fragments, such as oligogalacturonides, are typical DAMPs that elicit defense responses in many plants (Ridley et al., 2001; Sanabria et al., 2008; Ferrari et al., 2013) (Figure 1).

In the rice seedling apoplast, 45% of the proteins affected by H_2_O_2_ are involved in the carbohydrate and cell wall metabolism (Zhou et al., 2011). In this study, the abundance of most of the glycosylhydrolases such as α-galactosidases and β-1,3-glucanases, is found to be down-regulated. The authors suggested that the suppression of these polysaccharide hydrolases under H_2_O_2_ stress might reduce the hydrolysis of glucan and other polysaccharides altering the dynamic of remodeling of the polysaccharides to withstand the deleterious effects of oxidative stress. In comparison, the activities of α-L-arabinofuranosidases, UDP-glucose pyrophosphorylases and pectinesterases were up-regulated under H_2_O_2_ treatment suggesting that these enzymes might strengthen the cell wall by modulating polysaccharide degradation and synthesis, and increasing pectin demethylesterification. SA-treatment of *Arabidopsis* cell suspensions induces the accumulation of a large number of the proteins involved in general metabolism (34%) within the 6 first hours. Among them, α-galactosidase, α-1,4-glucan-protein synthase, pectinesterase, or β-fructofuranosidase are putatively involved in cell wall remodeling (Cheng et al., 2009). The fact that half of the identified secreted proteins are LSPs could partially explain the rapid extracellular accumulation of these proteins, which allows a rapid cell wall remodeling in response to defense signaling.

The agroinfiltration of tobacco leaves leads to the accumulation of a number of cell wall-modifying enzymes including galactosidases, α-L-arabinofuranosidases, β-D-xylosidases, peroxidases and proteases accounting for around 15% of the apoplastic proteins. These proteins are likely to be accumulated for cell wall maintenance and to complement constitutive defenses against bacterial pathogens (Goulet et al., 2010). *Verticillium longisporum* infection of *Arabidopsis* results in a specific increase of six extracellular proteins with overlapping functions in defense, development and cell wall metabolism (three peroxidases, germin, serine carboxypeptidase, α-galactosidase) (Floerl et al., 2012). The authors have correlated these changes in infected plants with a new synthesis of cell wall material with enhanced lignification and with a modification of metabolite contents.

Comparison of the apoplastic proteomes of *Arabidopsis* infected with virulent, avirulent or non-pathogenic strains of *Pseudomonas syringae* pv tomato revealed a strain-specific regulation of cell wall-modifying enzymes. For example, α-xylosidase accumulation is increased by MAMPs from the non-pathogenic mutant strain HrpA. By contrast, amount of the enzyme is decreased by an effector of the virulent strain DC3000, suggesting that this α-xylosidase could be important for *Pseudomonas syringae* resistance (Kaffarnik et al., 2009). In the same way, quantity of two glycosylhydrolases is specifically increased during the compatible *Arabidopsis*/*Pseudomonas syringae* interaction but not in the incompatible interaction with *Sinorhizobium meliloti*, demonstrating a microorganism-dependent regulation of these cell wall enzymes (De-La-Pena et al., 2008).

Out of the 700 proteins identified in the rice apoplast infected by *Magnaporthe oryzae*, 29 proteins from rice and 54 proteins from *Magnaporthe oryzae* were glycosylhydrolases (Kim et al., 2013). Moreover, 17 rice glycosylhydrolase genes were strongly activated at the transcriptional level after infection. Among these 17 genes, 4 glycosylhydrolases were expressed earlier or at a higher level in the incompatible interactions compared to the compatible ones. From the pathogen side, RT-PCR analysis revealed that the transcripts of 6 glycosylhydrolases families from *Magnaporthe oryzae* were differentially expressed in compatible interactions. Therefore, this study demonstrated that the extracellular modulation of both the pathogen and the host glycosylhydrolases have an important role in either promoting successful infection *via* the degradation of the host cell wall, or restricting the pathogen invasion through the reinforcement of the host defenses *via* the cell wall maintenance during early stages of infection.

### PR and other defense-related proteins

The defense-related proteins have various functions and are generally involved in different metabolisms other than defense. It is therefore relatively difficult to classify these proteins only in terms of their defense function. Some of these proteins such as peroxidases or oxalate oxidases have already been mentioned above. The defense-related proteins represent a large part of the basal apoplastic proteome including pathogenesis-related (PR) proteins, which are the most abundant (Delaunois et al., 2013). Unsurprisingly, the amount of the PR proteins is modulated in response to biotic stress in nearly all the secretome or apoplastic proteome studies listed in Tables 1, 2. However, despite the importance of PR proteins in plant defense, they generally only represent 10–15% of the proteins that are regulated in the apoplast following biotic stresses (Cheng et al., 2009; Kaffarnik et al., 2009; Zhou et al., 2011). The well-characterized chitinases degrade the cell walls of pathogen releasing PAMP-derived cell wall fragments that trigger MTI, thereby reinforcing the host defenses (Figure 1). Indeed, Kim et al. (2009) have identified up to nine chitinases induced by the rice blast fungus in the rice secretome. While chitinases are largely represented in the apoplast, not all of them are regulated in response to defense signaling. The analysis of the apoplastic proteomes of *Arabidopsis* infected with virulent or non-virulent strains of *Pseudomonas syringae* revealed that the effector of the virulent strain repressed two chitinases but only one is induced by MAMPs from the non-pathogenic strain (Kaffarnik et al., 2009). Similarly, two chitinases were identified in the *Arabidopsis* secretome in response to SA but only one was accumulated within the 2 first hours after treatment (Cheng et al., 2009). In response to H_2_O_2_, the rice apoplastic proteome analysis revealed the up-regulation of one chitinase and the down-regulation of two others (Zhou et al., 2011). All together, these results clearly indicate a pathogen- or signal-specific regulation of the chitinase pool in the apoplast.

Glucanases represent another large apoplastic protein family often co-induced with chitinases (Figure 1). Glucanases are known to limit fungal growth *via* the degradation of the glucans from fungal cell walls. *Verticillum longisporum* infection of *Brassica napus* induces the accumulation of one endochitinase and two β-1,3-glucanases (Floerl et al., 2008). *Agrobacterium* infiltration of tobacco leaves modulates the quantity of several chitinases and glucanases (Goulet et al., 2010). Moreover, the changes in the phosphorylation status of an endochitinase and an endo-1,4-β-glucanase revealed by the chitosan treatment of *Arabidopsis* cells (Ndimba et al., 2003) and the correlation of the transcript accumulation with the increase of acidic chitinases and β-1,3-glucanases in *Melampsora larici*/*Populus deltoides* interaction suggest a close regulation of these PR protein families (Pechanova et al., 2010). It should also be mentioned that glucanases could be involved in host cell wall remodeling putatively leading to the release of DAMPs molecules and thereby reinforcing the host defenses (Casasoli et al., 2008; Martinez-Esteso et al., 2009). Besides direct modifications that glucanases can produce in the cell wall, Finiti et al. (2013) suggested that they might interfere in the signaling network that operates during the defense response. Their enzymatic products, the β-1,3 glucans, can be considered as DAMPs and are known to be general elicitors of plant defense responses. The β-1,3 glucans were shown to induce variety of defense reactions in tobacco (Klarzynski et al., 2000), *Arabidopsis* (Ménard et al., 2004), or grapevine (Aziz et al., 2003, 2007), conferring resistance to viral, bacterial, and fungal pathogens. Moreover previous studies have demonstrated that the absence of the endoglucanases TomCel1 and TomCel2 in tomato and *Arabidopsis* alters the jasmonic acid signaling network limiting the necrotrophic pathogen *Botrytis cinerea* invasion and increasing the susceptibility to the hemibiotrophic *Pseudomonas syringae* DC3000 (Flors et al., 2007; Finiti et al., 2013). These results provide support for the contribution of endoglucanases in the establishment of the appropriate signaling response to pathogens by modifying the properties of the cell wall and/or interfering with signaling pathways.

Other PR proteins are regulated in the apoplast following biotic stress. The PR1 protein is accumulated in the apoplast of agroinfiltrated tobacco or H_2_O_2_-treated rice and the thaumatin-like protein accumulates in *Melampsora larici* infected poplar (Goulet et al., 2010; Zhou et al., 2011). Some lipases were also thought to act like PR proteins (Jakab et al., 2003). A lipase with a GDSL-like motif was identified in the grapevine secretome in response to JA (Martinez-Esteso et al., 2009) and in the *Arabidopsis* secretome in response to SA (Oh et al., 2005). The *Arabidopsis* lipase (GLIP1) was further characterized for its function in disease resistance and results suggest that GLIP1 may be a critical component in plant resistance. Indeed, the GLIP1 lipase disrupts the fungal spore integrity and triggers systemic resistance signaling in *Alternaria brassicicola* infected plants through the ET pathway (Oh et al., 2005).

Proteolytic enzymes in plants are directly or indirectly involved in most plant cellular processes including disease resistance (Xia et al., 2004). The induction of the amount of three subtilisin-like proteases, two aspartyl proteases, and one peptidase in the rice apoplast during *Magnaporthe oryzae* infection supports the view that secreted proteolytic enzymes might act as hydrolytic enzymes or mediators of signal transduction in the apoplast during pathogen attack (Kim et al., 2013). In this study, a total of 25 proteases/peptidase proteins from *Magnaporthe oryzae* were identified. These proteins are believed to play roles as pathogenicity factors required to circumvent the host defense responses. Therefore, the study of protease secretion in the apoplast is a promising resource for understanding some facets of plant-pathogen interactions.

Lectins are characterized by the presence of at least one jacalin-like domain that reversibly binds specific mono- or oligosaccharides. According to their carbohydrate specificities, plant lectins are important for a variety of biological processes including host–pathogen interactions. Specifically, they are believed to play a role in pathogen recognition and in plant defense responses (De Hoff et al., 2009). Several studies have shown their accumulation in the apoplast in response to SA treatment, chitosan or oligogalacturonides in *Arabidopsis* (Ndimba et al., 2003; Casasoli et al., 2008; Cheng et al., 2009) or in the rice-*Magnaporthe* interaction (Kim et al., 2009). Their regular identification in the apoplast under biotic-stress conditions reinforces their putative role in plant defense mechanisms.

In the rice-*Magnaporthe* interaction, the accumulation of several PR proteins is induced 72 h after infection in both the compatible and incompatible interactions but with a higher level in the incompatible interaction (Shenton et al., 2012). In the same plant-pathogen interaction, Kim et al. (2013) correlated the PR protein accumulation with upregulation of gene expression in both types of interactions and showed that three chitinase genes were expressed earlier or at a higher level in the incompatible interactions. Six proteins related to defense, such as peroxidases and basic chitinases were highly secreted in *Arabidopsis* 6 h after initial contact with *Pseudomonas syringae* but not in the incompatible interaction with *Sinorhizobium meliloti*, suggesting that *Arabidopsis* can selectively secrete defense proteins at an early stage of compatible interactions (De-La-Pena et al., 2008). Moreover, in the interaction between alfalfa with *Sinorhizobium meliloti* or *Pseudomonas syringae*, three chitinases, a thaumatin-like protein PR-5b, and a PR10-1 protein were secreted in abundance by alfalfa inoculated with *Sinorhizobium meliloti* at 6 h but were not secreted as much when it was inoculated with *Pseudomonas syringae*. The fact that alfalfa responds faster by secreting proteins in the presence of *Sinorhizobium meliloti*, but not in the presence of *Pseudomonas syringae*, suggests that an efficient signaling process similar to that operating during pathogenic interactions takes place during the early interaction with *Sinorhizobium meliloti*.

## Conclusions and future perspectives

Interest in the plant defense responses occurring in the apoplast is growing as the importance of this dynamic compartment becomes more apparent. The small number of studies indicates the limited availability of information on the potential role of the apoplastic proteome in plant-pathogen interactions. The first secretome analyses were reported using isolated MAMPs or signal molecule onto cell suspension cultures and, to our knowledge, only two secretome studies have been performed using intact pathogens (Table 1). A shift from cell-suspensions to *in planta* systems has taken place, but comparative disease-resistance studies are still scarce and little is known about the changes in the secretome during biotic stresses (Table 2). Based on the results of the proteomic studies reviewed here, our current understanding of biological processes occurring in the apoplast during plant–pathogen interactions is still rudimentary. Most of the proteins present in the apoplast are involved in the establishment of a basal defense in unstressed plants. Only a small number of these proteins are specifically modulated following the perception of a biotic stress.

Most of the studies listed in this review highlighted the regulation of the same families of proteins occurring in the apoplast during a biotic stress. The regulated proteins potentially involved in the mechanisms of perception and signal transduction such as DUF26 or LRR-like proteins appear to be less identified and/or characterized. The regulation of peroxidases, glucanases and chitinases is also emphasized since these large families of proteins are involved in the regulation of the cell redox status, the cell wall reorganization and the establishment of specific defenses (Figure 1). The general consensus suggests that accurate control of the speed and intensity of the protein secretion determines the establishment of effective resistance against a given pathogen.

The LSPs identified in most of the apoplastic proteomes in response to pathogen attacks may be one of the solutions used to increase the speed of protein secretion. Indeed, this secretory mechanism, independent of the classical ER-Golgi secretory pathway, could allow the rapid and efficient secretion of specific proteins providing a selective advantage in response to pathogen infection (Rose and Lee, 2010). Since most of the non-classically secreted proteins have established intracellular functions, it was suggested that they had dual roles with still unknown extracellular functions. There is growing evidence about the role of LSPs in plant defenses and the precise identification and characterization of these secreted proteins remain an exciting and challenging area of research.

Another way to increase the speed and specificity of the defense response in the apoplast may be the modulation of the PTMs of a pool of pre-proteins already present or the existence of several alternative PTMs affecting the final destination of the protein. Most of the apoplastic proteome studies in response to pathogen attacks suggested modulation of PTMs. Modification of PTMs could rapidly activate or repress the specific proteins involved in pathogen perception (like glycosylation) or signal transduction (like phosphorylation). It was demonstrated that the phosphorylation status of extracellular proteins rapidly changes in response to elicitor treatment, suggesting a possible role for the apoplastic proteins in early signal transmission of pathogen defenses through the activation of pathways regulated by external kinases and phosphatases (Casasoli et al., 2008). The establishment of plant phosphoproteomes has made remarkable progress and is now moving from qualitative to quantitative. However, more work needs to be done to investigate the precise phosphorylation nature and phosphorylation patterns. Moreover, there has been no global study of the glycosylation of apoplastic proteins upon pathogen infection. Plant glycoproteomics is only in its early stages but is a very promising approach toward an integrated study of both sugars and protein moities to gain new insight into the function of glycoproteins in plant defenses. Similarly, little is known about the oxidation of apoplastic proteins even if ROS and NO are important molecules playing key roles in apoplastic plant defenses. The study of PTMs is still in its initial phases, and although instrumentation and separation techniques can be improved, for many PTMs there are some existing methods available that can be adapted to plant disease proteomics research. Undoubtedly, future work needs to be directed toward a better understanding of the possible extracellular PTM events since the ability to define the dynamic proteome is crucial for unraveling novel mechanisms of plant-pathogen signaling.

It was suggested that secreted proteins might be a critical component in the process of signaling and recognition occurring between compatible and incompatible interactions. Infection of rice or *Arabidopsis* with an incompatible pathogen leads to a much earlier induction of genes and proteins than for a compatible interaction (Kaffarnik et al., 2009; Kim et al., 2013). These results highlight the importance of the early stages in the infection process and demonstrate the need for kinetic studies addressing complex organism interactions. More in-depth analyses of the spatial and temporal distribution of responding proteins will improve the understanding of pathogen invasion strategies and the complex interplay between hosts and pathogens. Future studies should focus on differential approaches based on compatible/incompatible interactions by using virulent/avirulent pathogen strains or sensitive/resistant host species. However, these studies should also include kinetics of apoplastic proteome and cell wall proteome at the early steps of the infection process to obtain an dynamic view of identified soluble and ionically bonded proteins from both the plant and the pathogen. Since a down-regulation of a protein upon pathogen attack might indicate regulation by pathogen effectors, functional analysis of a subset of identified secretory proteins from the pathogen implies that a number of them are likely to act as apoplastic effectors that can be recognized by receptors (Kim et al., 2013). As more and more evidence points to the biological role of the fungal effectors that manipulate plant immunity in favor of fungal virulence, the development of reliable quantitative proteomics will indeed be crucial to identifying putative effector targeting in the apoplastic proteome.

Thus, it is important to build a comprehensive inventory of the experimentally identified plant–pathogen secretome to predict secreted proteins more accurately, and then to address the question of their biological role. Apoplastic proteome analyses of plant–pathogen interactions have provided a better understanding of plant defense responses. However, the lack of published studies using quantitative and *in vivo* proteomic techniques is still striking. The improvement of peptide resolution sensitivity based on gel-free technology and the precise and absolute peptide quantification based on isotopic labeling approaches, such as iTRAQ technology, should greatly increase the number of identified apoplastic proteins upon pathogen challenge. The utility of absolute quantification of individual secreted proteins was clearly demonstrated in application to complex, time- and dose dependent experimental designs. There is also a need for performing more biological conditions rather than just technical replicates in experiments for quantification. Moreover, combining proteomic analyses with genetics and other omic approaches would strengthen the biological significance of many studies. A more systematic integration of these complementary approaches will provide useful information that will allow for better prediction and manipulation of plant responses to pathogens. Nevertheless, one of the main challenges in the near future will be to validate and explore the roles of individual secreted proteins involved in plant-pathogen interactions. While most proteomic studies provide protein identification and functional predictions, most of them do not test their hypotheses using genetics. Further studies will then be needed to assign functional roles to these secreted proteins in plant-pathogen interactions.

### Conflict of interest statement

The authors declare that the research was conducted in the absence of any commercial or financial relationships that could be construed as a potential conflict of interest.
